# Efficacy of therapeutic baths with *Piper callosum* essential oil against monogeneans of *Colossoma macropomum*: hematological and histopathological assessments

**DOI:** 10.1590/S1984-29612025031

**Published:** 2025-06-13

**Authors:** Carliane Maria Guimarães Alves, Raimundo Rosemiro Jesus Baia, Paulo Venicius Nascimento Santos, Amanda Mendes Pacheco, Marcela Nunes Videira, Francisco Célio Maia Chaves, Eliane Tie Oba Yoshioka, Marcos Tavares-Dias

**Affiliations:** 1 Programa de Pós-graduação em Biodiversidade Tropical – PPGBIO, Universidade Federal do Amapá – UNIFAP, Macapá, AP, Brasil; 2 Universidade do Estado do Amapá – UEAP, Macapá, AP, Brasil; 3 Programa de Pós-graduação em Ciências Ambientais, Universidade Federal do Amapá – UNIFAP, Macapá, AP, Brasil; 4 Embrapa Amazônia Ocidental, Manaus, AM, Brasil; 5 Embrapa Amapá, Macapá, AP, Brasil

**Keywords:** Blood, parasitic infection, fish freshwater, treatment, Sangue, infecção parasitária, peixe de água doce, tratamento

## Abstract

As chemotherapeutants used in control and treatment of monogeniosis can cause environmental damage and risks to human health, there is need of compounds alternatives effective and safe to this parasitosis. Essential oils are herbal compounds with good degradability in nature and effectiveness, and resistance absence that can be alternatives to chemotherapeutants. This study aimed to report the first trial on the effects of therapeutic baths with *Piper callosum* essential oil anti-monogeneans in fish. *Colossoma macropomum* (tambaqui) were subjected to six baths with 100 mg/L of *P. callosum* essential oil, each lasting 20 minutes per day (short duration), with 24-hour intervals between baths. Therapeutic baths demonstrated efficacy of 83.6% against monogeneans of tambaqui. Fish submitted to therapeutic baths had reduction statistically significant in the plasma levels of total proteins, similar to the control group exposed to water from culture tank + 70% alcohol. The toxicity of *P. callosum* essential oil was low as there were no significant blood or histopathological changes that compromised gill function in the treated fish. Due to high efficacy of 100 mg/L of *P. callosum* essential oil in controlling monogenean infestations, six short therapeutic baths on alternate days may be recommended as anti-monogeneans strategy for tambaqui.

## Resumo

Como quimioterápicos utilizados no controle e tratamento de monogeniose podem causar danos ambientais e riscos à saúde do homem, há necessidade de compostos alternativos eficazes e seguros para essa parasitose. Óleos essenciais são compostos herbais com boa degradabilidade natural e eficácia, e ausência de resistência que podem ser alternativas aos quimioterápicos. Este estudo teve como objetivo relatar o primeiro ensaio sobre os efeitos de banhos terapêuticos com óleo essencial de *Piper callosum* antimonogenéticos de peixes. *Colossoma macropomum* (tambaqui) foram submetidos a seis banhos com 100 mg/L de óleo essencial de *P. callosum*, durante 20 minutos/dia (curta duração), em intervalos de 24 horas entre eles. Os banhos terapêuticos tiveram 83,6% de eficácia contra monogenéticos das brânquias de tambaqui. Nos peixes submetidos aos banhos terapêuticos ocorreu diminuição nos níveis plasmáticos de proteínas totais, bem como no grupo controle exposto a água do tanque de cultivo + álcool. A toxicidade desse óleo foi baixa, pois não ocorreram alterações sanguíneas e histopatológicas significativas que poderiam comprometer o funcionamento das brânquias dos peixes. Devido à elevada eficácia de 100 mg/L de óleo essencial de *P. callosum* no controle de monogenéticos, seis banhos terapêuticos curtos, em dias intercalados, podem ser recomendados como estratégia anti-monogenéticos para tambaqui.

## Introduction

Monogeniosis is a parasitic disease caused by several species of monogenean helminths, which are harmful to different fish species worldwide. In global aquaculture, these parasites mainly affect the gills of fish, resulting in significant production losses due to severe infections (Tavares-Dias & Martins, 2017; Hoai, 2020; Mladineo et al., 2021), which can frequently be accompanied by opportunistic pathogens and increased stress (Silva et al., 2024; Alves et al., 2024a). Parasitosis caused by monogeneans can cause displacement of the gill epithelium, focal hyperplasia of epithelial cells, lamellar fusion, congestion, complete fusion of secondary gill lamellae, necrosis of the gill filaments due to damage to the gill epithelium, lethargy, opercular hyperactivity, hypoxia, and anemia caused by low hemoglobin levels (Tavares-Dias et al., 2021; Mladineo et al., 2021). Therefore, as the gills are a multifunctional organ, damage to their structure, such as infections caused by monogeneans, can interfere with homeostasis (Alves et al., 2024b).

In fish aquaculture, several chemicals have been used in therapeutic baths (e.g. formalin, hydrogen peroxide, potassium permanganate, albendazole, ivermectin, sodium chloride, levamisole, sodium peroxycarbonate, trichlorfon, copper sulfate, etc.) to control and treat monogeniosis. However, usually they are not safe to fishes due to their toxicity, present little efficacy and resistance and also can cause prejudice to environment due their toxicity; in addition are often economically unviable for the fish farmer (Alves et al., 2019; Hoai, 2020; Mladineo et al., 2021; Tadese et al., 2022; Silva et al., 2024; Morales-Serna et al., 2018), due to elevated cost of high concentration used in the tanks. Thus, there is a need to find new therapeutics alternatives such as the use of essential oils from medicinal plants for controlling and treating diseases caused by monogeneans (Hashimoto et al., 2016; Alves et al., 2021; Tadese et al., 2022), aiming at their application in fish farming.

Essential oils are composed of secondary metabolites of medicinal plants, have bioactive properties, and can be used as phytotherapeutic agents in sustainable aquaculture (Hashimoto et al., 2016; Alves et al., 2021, 2024a; Silva et al., 2024). *Piper callosum* Ruiz & Pav is a Piperaceae shrub native to Bolivia, Brazil, Peru, and Colombia. In Brazil, it occurs in the states of Acre, Amazonas, Amapá, Pará, Rondônia, Distrito Federal, Mato Grosso, Espírito Santo, Rio de Janeiro and Paraná. This medicinal plant is popularly known as ‘elixir paregórico’, ‘ventre-livre’, or ‘óleo elétrico’, and has been used as an aphrodisiac, astringent, digestive, antidiarrheal, and local hemostatic agent, to prevent leucorrhea, and to treat mosquito bites (Silva et al., 2017; Ayres et al., 2023). The major constituents of *P. callosum* essential oil are safrole (53.8%), α-pinene (12.2%), methyl eugenol (7.6%), and 1,8- cineole (3.7%) (For more details, see Alves et al., 2021).

Recently, concentrations of 600 to 2,000 mg/L of *P. callosum* essential oil showed 100% *in vitro* efficacy against the monogeneans *Anacanthorus spathulatus* Kritsky, Thatcher & Kayton 1979; *Notozothecium janauachensis* Belmont-Jégui, Domingues & Martins 2004; *Mymarothecium boegeri* Cohen & Kohn, 2005 and *Linguadactyloides brinkmanni* Thatcher & Krytsky, 1983 in gills of *Colossoma macropomum* Cuvier 1818 (Alves et al., 2021), the most farmed native fish in Brazil (Alves et al., 2024b; PeixeBR, 2025). Therapeutic baths with essential oil of *Piper hispidum* Swartz also showed high efficacy against these same species of monogeneans in gills of *C. macropomum* (Alves et al., 2024a), while baths with *Piper marginatum* Jacq had low efficacy in controlling these parasites (Alves et al., 2024b). Efficacy *in vitro* and *in vivo* of seed extracts from *Piper guineense* Schumach & Thonn against monogeneans *Gyrodactylus elegans* von Nordmann 1832 and *Dactylogyrus extensus* Mueller & Van Cleave, 1932 in *Carassius auratus* Linnaeus, 1758 has been studied. A high efficacy (100%) *in vitro* was observed, while no efficacy *in vivo* was found in the gills of this fish host (Ekanem et al., 2004). However, the efficacy of therapeutic baths with the essential oil of *P. callosum* has not been evaluated against monogeneans of fish.

Therefore, a major challenge in the search for sustainable aquaculture is maintaining fish health against diseases caused by monogeneans, and the use of natural compounds is a current requirement. Thus, as there are no *in vivo* studies with exposure to *P. callosum* essential oil, we investigated the efficacy of therapeutic baths with this essential oil against monogeneans of *C. macropomum* gills, as well as the possible effects of this exposure in hematology and histopathology of this host fish.

## Material and Methods

### Fish, acclimation and parasites

*Colossoma macropomum* juveniles were obtained from a commercial fish farm in Macapá (00°08'42.3”N-051°06'04.2”W), state of Amapá, Brazil, and maintained at the Aquaculture and Fisheries Laboratory of Embrapa “Laboratório de Aquicultura e Pesca da Embrapa Amapá”, Macapá, Amapá State, Brazil. These fish were acclimated in 500 L tanks and kept in an open water system with constant aeration and continuous water renewal (1.1 L/min) for 10 days and fed twice daily with a commercial diet containing 34% crude protein (Guabi^®^, Brazil). This fish stock naturally infected with monogeneans was used in all assays.

The following water parameters were measured daily: mean temperature (30.8 ± 0.1°C), dissolved oxygen (5.6 ± 0.2 mg/L), pH (5.9 ± 0.2), ammonia (0.4 ± 0.1 mg/L), alkalinity (11.0 ± 0.001 mg/L) and hardness (11.0 ± 0.1 mg/L), using a multiparameter probe (YSI, USA). The tank was siphoned weekly to remove accumulated organic matter, and the water was replenished.

### Therapeutic baths in *C. macropomum* with essential oil of *P. callosum*

In 2024 July, juveniles of *C. macropomum* with weight of 49.7 ± 6.9 g and total length of 15.0 ± 0.9 cm (31.0-68 g and 1.7-18.7 cm) were subjected to therapeutic baths with 100 mg/L of *P. callosum* essential oil for 20 minutes per day, at 24-hour intervals, for six days, using three treatments with three replications each (13 fish per replication), totaling 39 fish per treatment. The fish were kept in a static water system upon the addition of the essential oil in the experimental tanks. After this, the water in the tanks was changed.

The essential oil of *P. callosum* was diluted daily with 70% ethyl alcohol (1:10 w/v) before being added to the tank water, to obtain 100 mg/L. Two control groups were used: one with water from the culture tank, and the other with water from the culture tank + ethyl alcohol (70%). On the sixth day following the therapeutic baths, a total of 90 fish (30 fish by treatment) were euthanized by medullary section and the gills were collected and fixed in 5% formalin to count the monogeneans (Eiras et al., 2006) and determine the prevalence and average abundance of these parasites (Bush et al., 1997). The efficacy of the therapeutic baths was determined using calculations described by Wang et al. (2008).

For the therapeutic baths with 100 mg/L of the *P. callosum* essential oil, *C. macropomum* were submitted to previous studies of tolerance (i.e., maximum concentration of essential oil supported by the fish) with this essential oil (for more details, see Alves et al., 2021).

### Blood analysis in *C. macropomum* after therapeutic baths with *P. callosum* essential oil

After six days of therapeutic baths with 100 mg/L of *P. callosum* essential oil, fish blood was collected by puncturing the caudal vein using syringes with ethylenediamine tetra-acetate (10% EDTA). Three treatments with three replications of five fish each, totaling 15 fish per treatment, were used. Two control groups were used: one with water from the culture tank, and the other with water from the culture tank + ethyl alcohol (70%). The blood was analyzed for hematocrit, using the microhematocrit method, counts of the total number of erythrocytes in a Neubauer chamber, and hemoglobin concentration by the cyanmethemoglobin method. Hematimetric indices such as mean corpuscular volume (MCV) and mean corpuscular hemoglobin concentration (MCHC) were calculated from the data of erythrocytes, hematocrit, and hemoglobin (Ranzani-Paiva et al., 2022).

### Histopathological analysis of *C. macropomum* gills after therapeutic baths with *P. callosum* essential oil

On the sixth day after the therapeutic baths with 100 mg/L of *P. callosum* essential oil, the fish were euthanized by medullary section. The gills of nine animals from each treatment (three from each replication) were collected for histopathological analysis. The first right and left gill arch of each fish were collected and fixed in Davidson's solution (95% alcohol, formaldehyde, acetic acid, and distilled water) for 48 hour, then dehydrated in an increasing alcohol series (70%, 80%, 90%, absolute I, II and III), cleared in xylene (100%), impregnated and embedded in paraffin to obtain the blocks.

The paraffin blocks were sectioned at 5 µm thickness using a microtome (Leica DM 1000). After mounting the slides (in duplicates), they were stained with Hematoxylin and Eosin (HE). Images were captured with a digital camera (Moticam 2300 3.0 M Pixel) attached to a common optical microscope, and connected to the computer containing the image capture software. The histopathological analyses were performed semiquantitatively, using the mean assessment values (MAV) (Schwaiger et al., 1997) and histopathological alteration index (HAI) (Poleksić & Mitrović-Tutundžić, 1994).

### Statistical analysis

The histopathological, blood, and parasitic data were previously subjected to analysis to assess normality, using the Shapiro-Wilk test, and the “RVAideMemoire” package (Herve, 2023), and homoscedasticity, using the Levene test from the “car” (Fox & Weisberg, 2019). All data showed homogeneity in variance. Histopathological and blood data showed non-normal distribution, while parasite abundance data showed normal distribution. In this way, the Kruskal-Wallis test was adopted to evaluate the effect of treatments on the histopathological and blood parameters of the animals, followed by the Dunn post hoc test using the “rstatix” package (Kassambara, 2023). For parasite data, the ANOVA standard “aov” function of the R software was used, which aims to evaluate the effect of treatments on the abundance of monogeneans, followed by Tukey's post hoc test using the “DescTools” package (Signorell, 2023). All these analyses were run in the R software (R Core Team, 2022).

## Results

Over the period of the six therapeutic baths with 100 mg/L of *P. callosum* essential oil, there was no mortality of fish in any of the treatments. The behaviors observed in the fish were: agitation, accelerated opercular movements, lethargy, and erratic swimming, caused by the sedative effects of the *P. callosum* essential oil used in the therapeutic baths. During the treatments, the fish were fed and ate normally in all experimental groups.

All fish (100%) used in therapeutic baths presented their gills naturally parasitized by three species of monogeneans (*A. spathulatus*, *M. boegeri* and *N. janauachensis*). In the gills of *C. macropomum* submitted to therapeutic baths with 100 mg/L of *P. callosum*, the abundance of monogeneans was lower (p<0.05) than in control group exposed to culture tank water and control group water from the culture tank + 70% alcohol, which were similar (p>0.05) to each other. However, parasite prevalence was similar among all experimental treatments. Therapeutic baths with 100 mg/L of *P. callosum* essential oil showed high efficacy anti-monogeneans in *C. macropomum* gills. The control group with culture tank water + 70% alcohol also had some efficacy, but it was minimal (Table 1).

**Table 1:** Efficacy and parasitological indices of monogeneans in gills of *Colossoma macropomum* submitted to therapeutic baths with *Piper callosum* essential oil.

**Treatments**	**Prevalence (%)**	**Mean abundance**	**Dunn test**	**Efficacy (%)**
Water	100	30.8 ± 10.4^a^	63.5	-
Water + alcohol	100	26.8 ± 8.8^a^	57.4	12.8
100 mg/L	100	4.4 ± 1.6^b^	15.5	83.6

Data express mean ± deviation standard. Different letters, in the same column, indicate differences by the Dunn test (p<0.05).

In fish submitted to six therapeutic baths with 100 mg/L of *P. callosum* essential oil and the control group with culture tank water + 70% alcohol, the plasma levels of total proteins were lower (p<0.05) than in the control group with culture tank water. However, the MCV of fish exposed to 100 mg/L of *P. callosum* essential oil increased (p<0.05) compared to both controls, while the other blood parameters were not altered (Table 2).

**Table 2:** Blood parameters of *Colossoma macropomum* submitted to therapeutic baths with *Piper callosum* essential oil.

**Parameters**	**Water**	**Water + alcohol**	**100 mg/L**
Glucose (mg/dL)	70.0 ± 21.2^a^	86.6 ± 26.3^a^	88.1 ± 23.5^a^
Total protein (g/dL)	2.9 ± 0.5^a^	1.9 ± 0.7^b^	1.7 ± 0.3^b^
Erythrocytes (x10^6^/μL)	0.92 ± 0.13^a^	0.95 ± 0.10^a^	0.68 ± 0.09^a^
Hematocrit (%)	24.5 ± 4.2^a^	23.5 ± 2.0^a^	24.4 ± 2.0^a^
Hemoglobin (g/dL)	7.7 ± 1.4^a^	7.2 ± 1.0^a^	7.8 ± 1.6^a^
MCV (fL)	273.5 ± 63.3^a^	250.3 ± 21.2^a^	364.8 ± 63.0^b^
MCHC (g/dL)	31.4 ± 2.4^a^	30.7 ± 3.6^a^	31.9 ± 5.6^a^

Data express mean ± deviation standard. Different letters, in the same row, indicate differences by the Dunn test (p<0.05). MCHC: Mean corpuscular hemoglobin concentration; MCV: Mean corpuscular volume.

**Figure 1:**
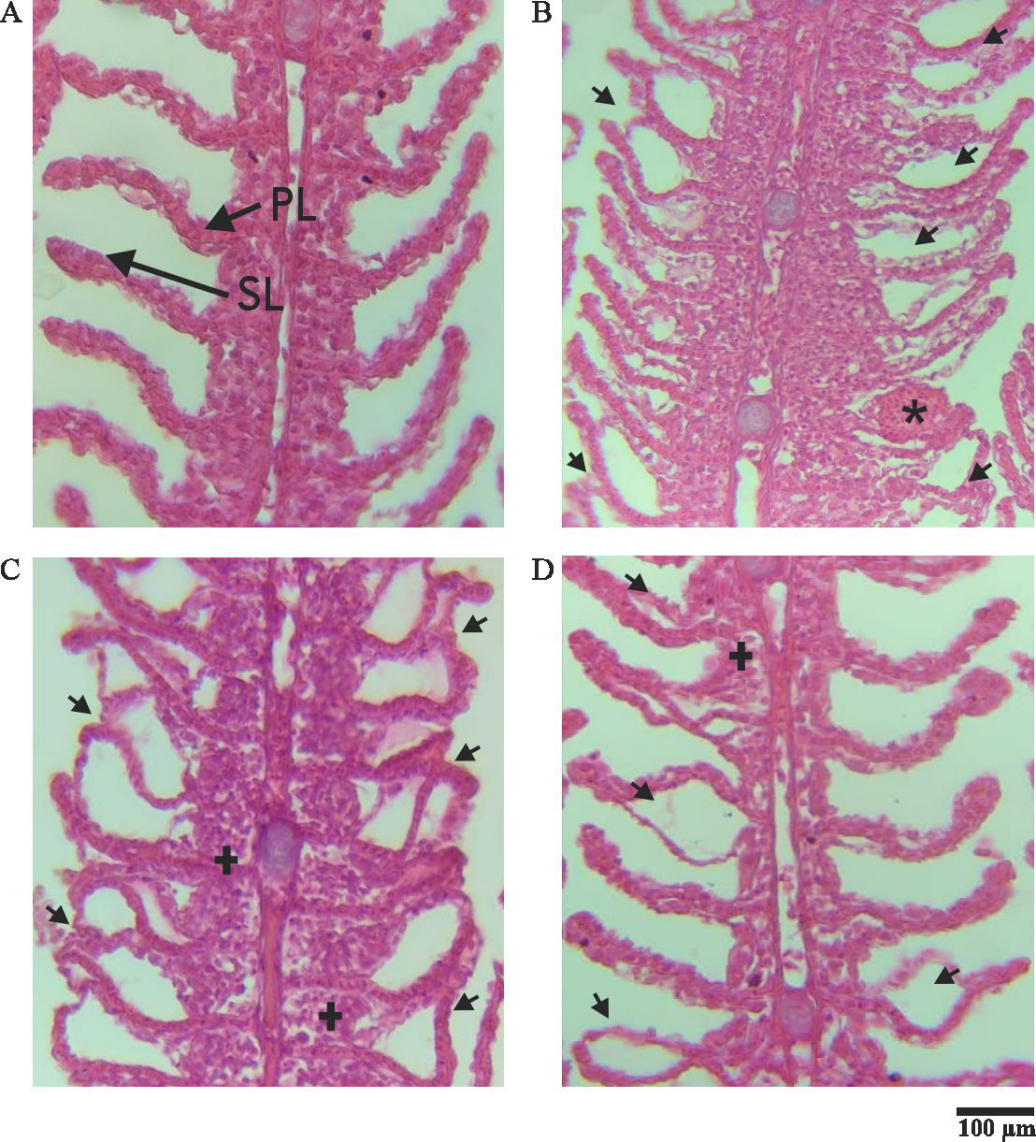
Histopathology of the gills of *Colossoma macropomum* submitted to therapeutic baths with 100 mg/L of *Piper callosum* essential oil. (A) Fish gills exposed to culture water (control) showing primary (PL) and secondary (SL) lamellae (arrow); (B-C) Fish gills exposed to water + 70% alcohol (control). B. Gill filaments showing detachment of the lamellar epithelium (arrow) and aneurysm-like lesions of lamellar blood vessels (asterisk); (C) Branchial filaments showing lamellar hyperplasia (+) and detachment of the lamellar epithelium (arrow); (D) Fish gills exposed to 100 mg/L of *Piper callosum*, gill filaments showing detachment of the lamellar epithelium (arrow).

In the gills of control fish exposed only to culture tank water, there were no notable histopathological changes. In contrast, in those exposed to culture tank water + 70% alcohol, the gill filaments showed detachment of the lamellar epithelium, aneurysm of the lamellar blood vessels, and lamellar hyperplasia. In the gills of fish subjected to therapeutic baths with 100 mg/L of *P. callosum*, the gill filaments showed hyperplasia and detachment of the lamellar epithelium (Figures 1A-D). After therapeutic baths with 100 mg/L of *P. callosum* essential oil, the histopathological analysis of the gills showed no significant differences (p>0.05) in HAI and MAV compared to the treatment with essential oil and the control groups with culture tank water and with culture tank water + 70% alcohol (Table 3).

**Table 3:** Values of histopathological alteration index (HAI) and mean assessment values (MAV) for gills of *Colossoma macropomum* submitted to therapeutic baths with *Piper callosum* essential oil.

**Treatments**	**N**	**MAV**	**HAI**	**Severity of the lesions according to the HAI**
Water	9	3.0 ± 1.0^a^	5.6 ± 5.0^a^	Normal functioning of the organ
Water + alcohol	9	4.0 ± 1.6^a^	6.5 ± 5.7^a^	Mild to moderate organ damage
100 mg/L	9	3.0 ± 1.2^a^	5.2 ± 4.7^a^	Normal functioning of the organ

Data express mean ± deviation standard. Different letters, in the same column, indicate differences by the Dunn test (p< 0.05).

## Discussion

Phytotherapeutics in fish aquaculture may present several advantages, such as reduced environmental impact, biodegradability, lower residue levels in fish muscle and lower toxicity to host fish; without causing parasitic resistance (Tadese et al., 2022; Silva et al., 2024), in addition of therapeutic efficacy anti-monogeneans in fish (Ekanem et al., 2004; Alves et al., 2024a; Silva et al., 2024). Hence, the use of essential oils has been a growing alternative in the control and treatment of infections caused by monogeneans in fish aquaculture (Alves et al., 2024a, b).

However, up to date, studies on the efficacy of therapeutic baths with *P. callosum* in fish are scarce. In the present study, six therapeutic baths with 100 mg/L of *P. callosum* essential oil showed 83.6% efficacy against monogeneans of *C. macropomum* gills. Similarly, baths with 100 mg/L of *P. hispidum* essential oil showed 78.6% therapeutic efficacy against monogeneans infesting the gills of *C. macropomum* (Alves et al., 2024a). Conversely, therapeutic baths using 100 mg/L of *P. marginatum* were ineffective against gill parasites in *C. macropomum* (Alves et al., 2024b). Although the same strategies were adopted in the therapeutic baths of these studies, this variation in efficacy arises from differences in the composition of major constituents of these essential oils (Alves et al., 2021), which can have distinct effects anti- monogeneans.

Fish blood carries various constituents such as nutrients, hormones, minerals, proteins, immunological components, etc. Total protein levels are one of the most common and useful blood parameters to determine in fish. Proteins perform a wide range of functions, including maintaining osmotic pressure, and pH, transporting various metabolites, and closely interacting with the immune system, as they play a key role in fish humoral immunity and innate immune response (Esmaeili, 2021). In *C. macropomum* subjected to therapeutic baths with 100 mg/L of *P. callosum* essential oil and control group with tank water + 70% alcohol, levels of plasma total protein decreased than compared to the control group with tank water. Likewise, total protein levels were reduced after exposure of *C. macropomum* to therapeutic baths with 100 mg/L of *P. marginatum* (Alves et al., 2024b). On the other hand, after therapeutic baths with 100 mg/L of *P. hispidum*, no change in total protein levels in *C. macropomum* was reported by Alves et al. (2024a). Therefore, as only levels of total proteins were determined herein, further studies with other biomarkers such as leukocyte number, liver enzymes levels or immune gene expression are necessary for indicate the involvement of the immune system with the exposure of *P. callosum* essential oil.

Red blood cells number, hematocrit, and hemoglobin concentration are blood parameters that can be used to diagnose anemia in fish populations, thus indicating their health status (Ranzani-Paiva & Tavares-Dias, 2024). These erythrocyte parameters can be affected if the gills are compromised. Therefore, these can be indicators of the oxygen transport capacity of fish, thus relating to the concentration of oxygen available in the aquatic environment (Esmaeili, 2021; Ranzani-Paiva & Tavares-Dias, 2024). Changes in hematocrit caused by stress can lead to hemoconcentration or hemodilution. The first can be due to the loss of fluids by the body or the release of erythrocytes from the spleen, increasing hematocrit values. Hemodilution can be associated with some pathologies, leading to a reduced number of red blood cells, hemoglobin content, and hematocrit levels (Esmaeili, 2021; Ranzani-Paiva & Tavares-Dias, 2024). However, in *C. macropomum*, after six days of therapeutic baths with 100 mg/L of *P. callosum* essential oil, there was only an increase in MCV, indicating the presence of larger erythrocytes in these fish. Nevertheless, in *C. macropomum* subjected to therapeutic baths with 100 mg/L of *P. marginatum* was reported only increase of hematocrit (Alves et al., 2024b).

In fish, gills play a fundamental role in respiration, as they protect the filaments and absorb oxygen dissolved in water, thus allowing the constant flow of this gas through the respiratory epithelium, in addition to enabling the elimination of carbon dioxide from the blood, which is brought from the tissues and pumped by the heart to the gills. Besides the respiratory gas exchange, fish gills are responsible for ion absorption and acid-base transport, among other essential functions for fish (Schwaiger et al., 1997; Machado, 1999; Tavares-Dias et al., 2021; Chen et al., 2023). In the gills of *C. macropomum*, in the present study, exposed to water from the culture tank + 70% alcohol, there was a detachment of the lamellar epithelium, aneurysm of the lamellar blood vessels, and lamellar hyperplasia. These lesions varied from mild to moderate, which little affected the functioning of this respiratory organ. In the gills of fish subjected to six therapeutic baths with 100 mg/L of *P. callosum*, hyperplasia and detachment of the lamellar epithelium were observed. Alcohol causes inhibition on total ATPase, Na^+^/K^+^ ATPase, Ca^2+^ ATPase and Mg^2+^ ATPase activity in the gills of fish (Bhanu & Philip, 2011). Similar results were reported in the gills of C. *macropomum* after therapeutic baths with 100 mg/L of *P. maginatum* (Alves et al., 2024b) and 100 mg/L of *P. hispidum* (Alves et al., 2024a). However, in the fish hosts of present study, there were no changes in the gills of fish in the control group exposed only to water from the culture tank, in contrast to what was described by Alves et al. (2024a) for C. *macropomum*; although in both studies the abundance of monogeneans had been similar.

In conclusion, six therapeutic baths with 100 mg/L of *P. callosum* essential oil presented low toxicity to *C. macropomum* and good anthelmintic efficacy anti-monogeneans of the gills of this fish host. Under the conditions and concentration tested, this essential oil was safe for *C. macropomum* since hematological findings showed few changes and the gills were little affected by the treatment. Lastly, while the results with this concentration of *P. callosum* essential oil have been promising for use in the control and treatment of infections caused by monogeneans in *C. macropomum* aquaculture; there is still a need for validation of these on field, in addition complementary studies with biomarkers related to the status of the immune system of *C. macropomum* after exposure.
